# Supplementary material for: A recyclable biomass electrolyte towards green zinc-ion batteries
Source: Nat Commun. 2023 Jul 22;14:4435. doi: 10.1038/s41467-023-40178-0 (PMC10363112; doi:10.1038/s41467-023-40178-0)
Supplement: Supplementary file 1 — Supplementary Information [file 41467_2023_40178_MOESM1_ESM.pdf]

Supplementary information for

A Recyclable Biomass Electrolyte Towards Green Zinc-Ion Batteries

Hongyu Lu,[1,2†] Jisong Hu,[3†] Xijun Wei,[1†] Kaiqi Zhang,[4†] Xiao Xiao,[1] Jingxin Zhao,[5*] Qiang Hu,[6] Jing Yu,[7] Guangmin Zhou,[1*] Bingang Xu[5*]

[1]Tsinghua Shenzhen International Graduate School, Tsinghua University, Shenzhen 518055, P. R. China

[2]State Key Laboratory of Advanced Welding and Joining, School of Materials Science and Engineering, Harbin Institute of Technology, Harbin 150001, P. R. China

[3]School of Optical and Electronic Information, Huazhong University of Science and Technology, Wuhan 430074, P. R. China

[4]School of Marine Science and Technology, Harbin Institute of Technology (Weihai), Weihai 264209, P. R. China

[5]Nanotechnology Center, School of Fashion and Textiles, The Hong Kong Polytechnic University, Hung Hom, Kowloon, Hong Kong 999077, P. R. China

[6]School of Materials and Energy, University of Electronic Science and Technology of China, Chengdu 610054, P.R. China

[7]School of Physics, Harbin Institute of Technology, Harbin 150001, P. R. China

* Corresponding authors: Guangmin Zhou (Email: guangminzhou@sz.tsinghua.edu.cn); Jingxin Zhao (Email: jingxzhao@polyu.edu.hk); Bingang Xu (tcxubg@polyu.edu.hk) † These authors were equal major contributors

1.Supplementary Experimental Details

1.1 Calculation Details. The specific capacity of Zn/CPZ-H//MnO_2 full cells are acquired from the discharge curve of GCD tests based on the following equations:

$$C = \frac{\int_0^{\Delta t} idt}{m}$$

Where C (mAh g) is the specific capacity of full cell, i (mA) indicates discharge current, Δt (h) is the discharge time, and m (mg) represents the mass of the active material on the electrodes.

The energy density of the Zn/CPZ-H//MnO_2 full cell was calculated based on the following equation:

$$E = \frac{C \times m_{MnO_2} \times V}{m_{MnO_2}}$$

Where C indicates the calculated specific capacity, V is the operating voltage, m_{MnO2} is the mass of the active material on MnO_2 cathode.

The power density of the Zn/CPZ-H//MnO_2 full battery was calculated by the following equation:

$$P = \frac{E}{t}$$

Where E is the calculated energy density and t is the discharge time.

The contribution of capacitance behavior of full cell can be qualitatively calculated by the following equations:

$$i = av^b$$

Which can be expressed as:

$$\log(i) = b\log(v) + \log(a)$$

Where i is the peak current (A), v is the relevant scan rate (V s^{-1}), a and b are adjustable parameters. The b value can be acquired from the linear relation between log (i) and log (v) of the redox peaks. When b = 0.5,capacitance behavior is dominated by diffusion-controlled. The value of b approaches 1.0, capacitance behavior is dominated by capacitive energy storage process.

The contribution of capacitance process and diffusion process in whole capacity can be calculated by the following formula:

$$i = k_1v + k_2v^{1/2}$$

Which can be reformulated as:

$$i/v^{1/2} = k_1v^{1/2} + k_2$$

Where i is the current response, k_1v is the capacitance contribution, and $k_2v^{1/2}$ indicates diffusion-controlled contribution. The value of k_1 can be calculated.

The depth of discharge (DOD) of Zn anode was calculated by the following equation:

$$DOD_{(\%)} = \frac{I \times t \times S}{m \times C_{theoretical}} \times 100\%$$

Where I (mA cm^{-2}) indicates the applied current density, t (h) is the discharge time, m(g) represents mass of electrode, S(cm^2) is actual area of electrode, $C_{theoretical}$ is theoretical specific capacity of Zn anode (~820 mAh g^{-1}).

Specifically,

$$DOD_{(5\%)} = \frac{4.1\ mA\,cm^{-2} \times 1\ h \times 1\ cm^{-2}}{0.05\ g \times 820\ mAh\,g^{-1}} \times 100\%$$

$$DOD_{(25\%)} = \frac{10.25\ mA\,cm^{-2} \times 1\ h \times 1\ cm^{-2}}{0.05\ g \times 820\ mAh\,g^{-1}} \times 100\%$$

$$DOD_{(40\%)} = \frac{16.4\ mA\,cm^{-2} \times 1\ h \times 1\ cm^{-2}}{0.05\ g \times 820\ mAh\,g^{-1}} \times 100\%$$

$$DOD_{(80\%)} = \frac{32.8\ mA\,cm^{-2} \times 1\ h \times 1\ cm^{-2}}{0.05\ g \times 820\ mAh\,g^{-1}} \times 100\%$$

2. Supplementary Notes

2.1 Characterization of MnO_2 cathode.

MnO_2 cathode materials were synthesized by a simple one-step hydrothermal method. Field-emission scanning electron microscope (SEM) images (Supplementary Figure15a, b) show that the as-obtained MnO_2 possesses a ball-like structure composed of MnO_2 nanosheets with 300-500 nm, which exhibits a hierarchical porous structure. The transmission electron microscopic (TEM) images of the as-obtained MnO_2 samples (Supplementary Figure 15c) show a morphology similar to that observed in the SEM images, and this highly porous structure may be accessible to electrolytes with a better capacitance properties. A typical high-resolution TEM image (Supplementary Figure 15d) shows well-defined lattice fringes with a spacing of 0.31 nm, which agrees well with the crystallographic plane spacing (110) of MnO_2.

3. Supplementary Figures

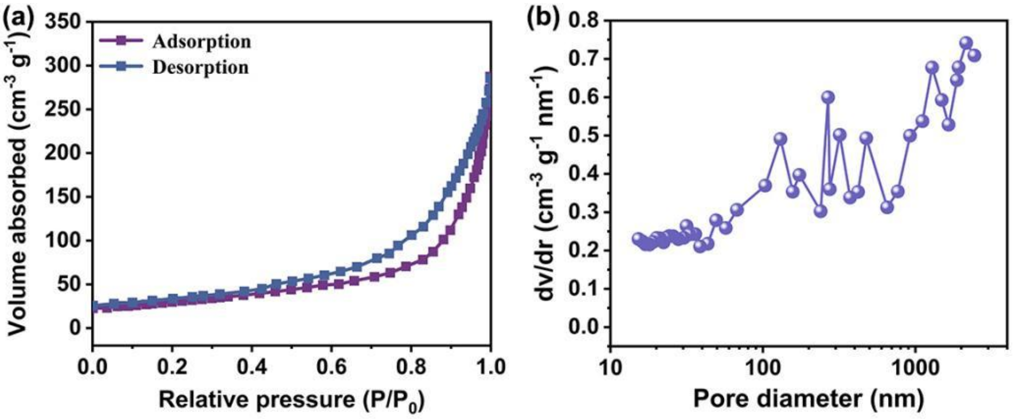

Supplementary Figure 1. Measurement of specific surface area of CPZ-H. (a) N_2 absorption/desorption isotherms and the pore size distribution (b) of the freeze-dried CPZ-H electrolyte.

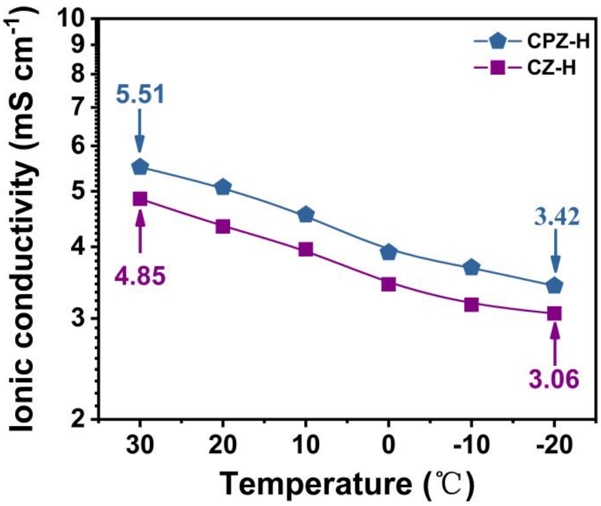

Supplementary Figure 2. Ionic conductivity of CPZ-H and CZ-H electrolytes at different temperatures.

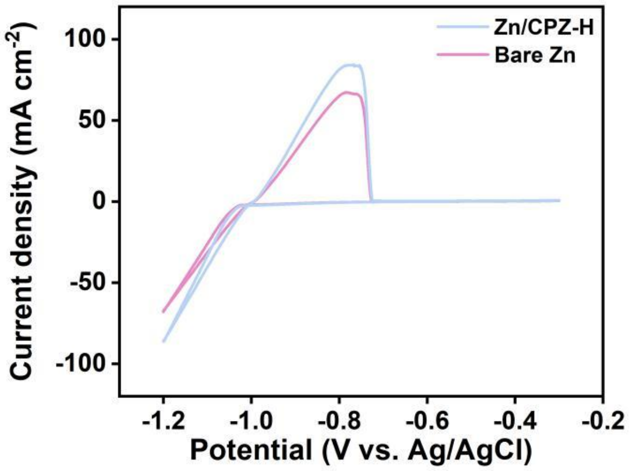

Supplementary Figure 3. CV curves of Zn/CPZ-H and bare Zn anodes.

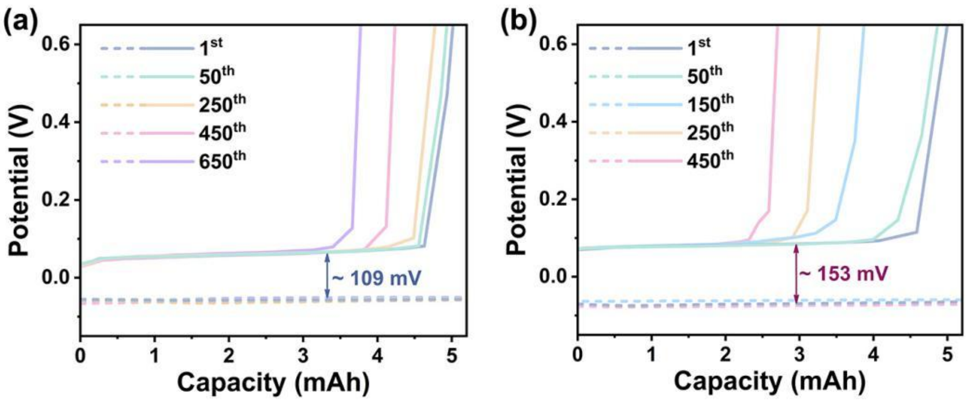

Supplementary Figure 4. Voltage profiles of the (a) Zn/CZ-H and (b) bare Zn anodes at 10 mA cm^{-2}.

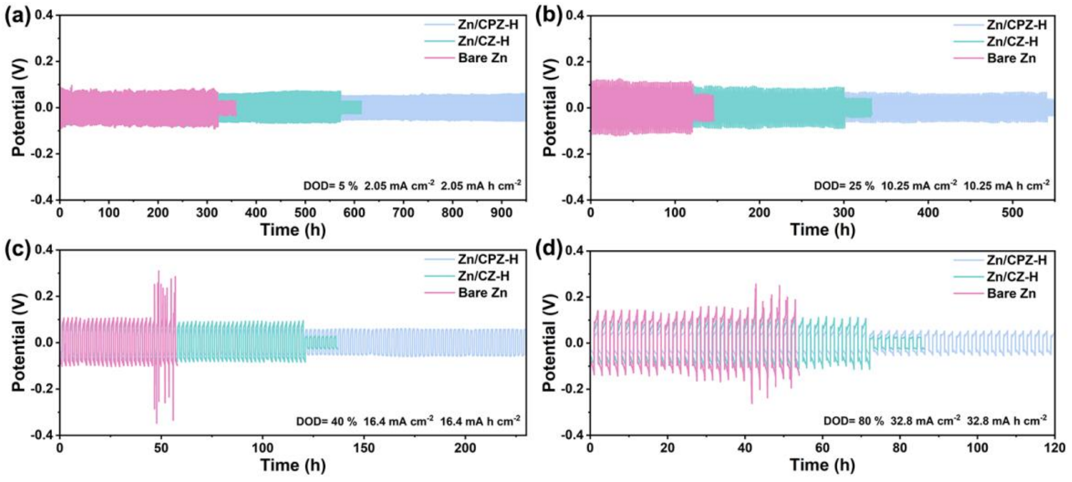

Supplementary Figure 5. Long-term galvanostatic cycling of symmetrical Zn cells at high (a) DOD:5 %, (b) 25 %, (c) 40 % and (d) 80 %.

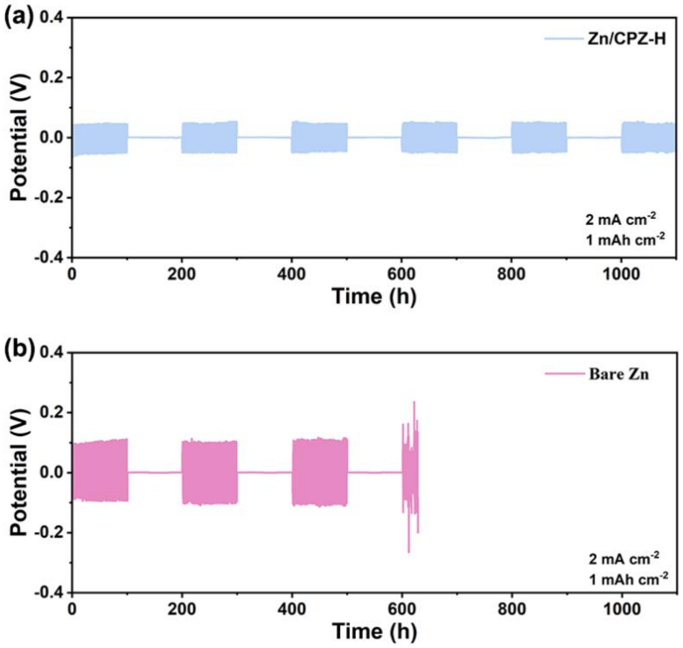

Supplementary Figure 6. Shelving recovery performance of (a) bare Zn/CPZ-H and (b) bare Zn symmetric cells.

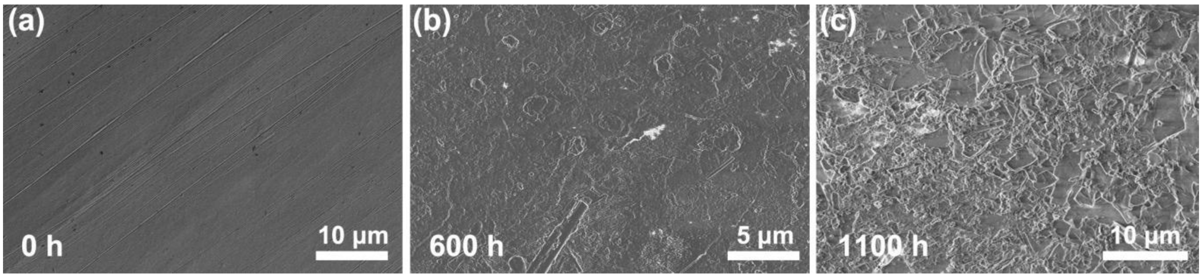

Supplementary Figure 7. Ex-situ SEM images of Zn/CZ-H anode at (a) 0 h, (b) 600 h and (c)1100 h.

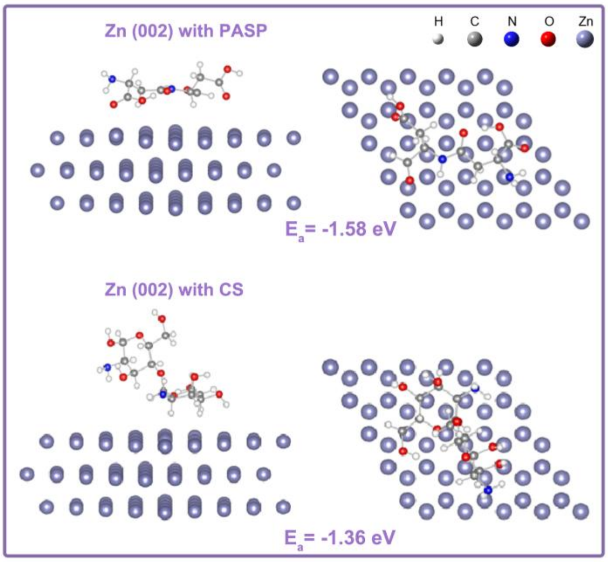

Supplementary Figure 8. Adsorption energy of the PASP and CS to Zn (002) plane.

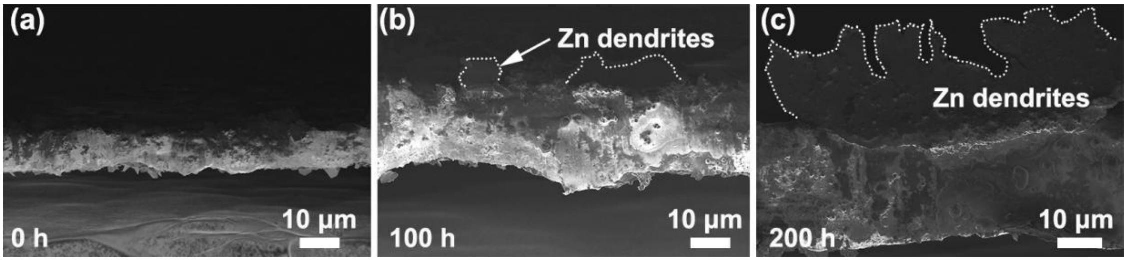

Supplementary Figure 9. Cross-sectional SEM images of bare Zn anode at cyclic time of (a) 0 h, (b) 100 h and (c) 200 h.

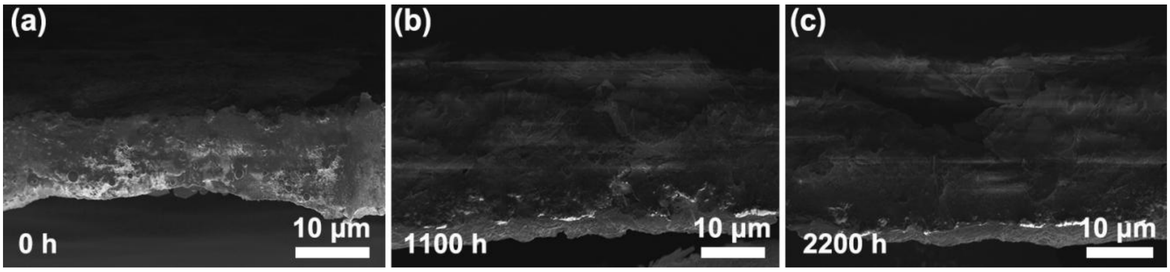

Supplementary Figure 10. Cross-sectional SEM images of Zn/CPZ-H anode at cyclic time of (a) 0 h, (b) 1100 h and (c) 2200 h.

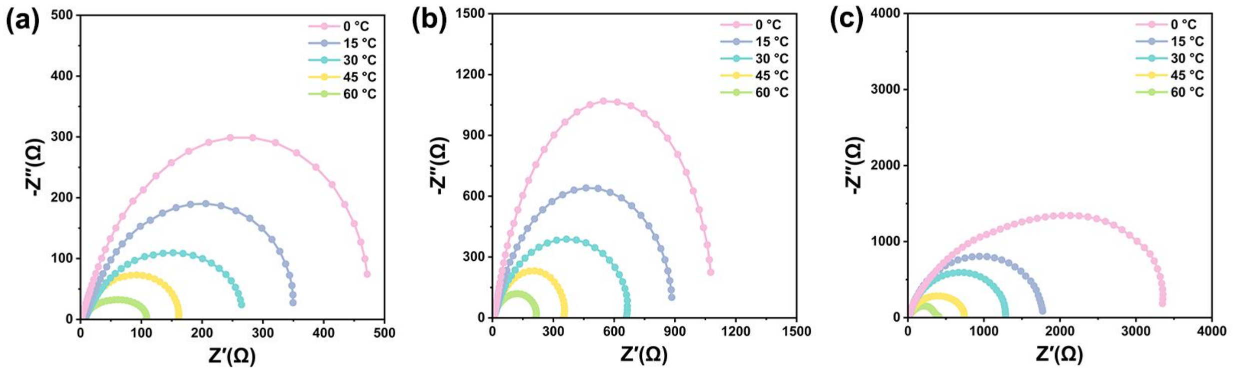

Supplementary Figure 11. Nyquist plots at different temperatures for (a) Zn/CPZ-H, (b) Zn/CZ-H and (c) bare Zn symmetric cells.

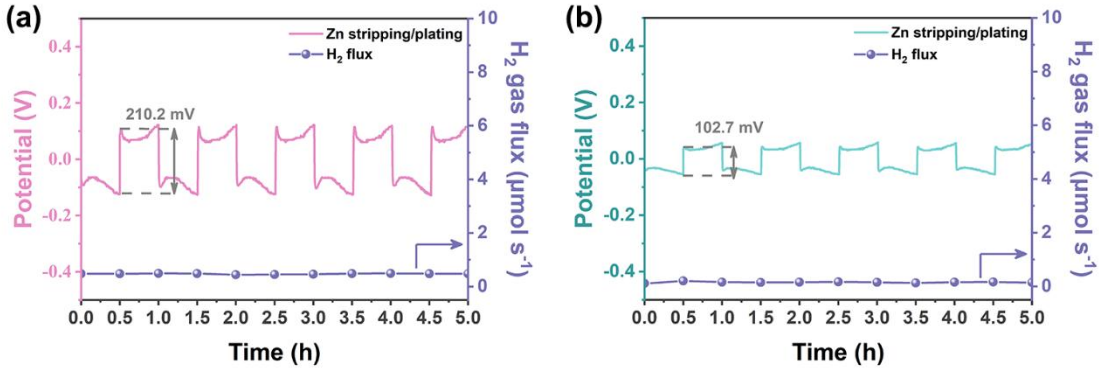

Supplementary Figure 12. Ex-monitoring of H_2 evolution flux of (a) bare Zn anode and (b) Zn/CZ-H anode.

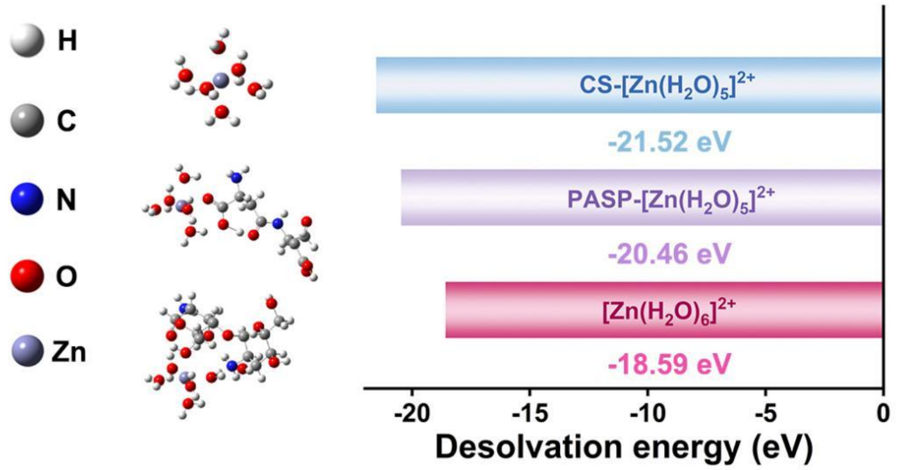

Supplementary Figure 13. Calculated desolvation energies of CS–$[Zn(H_2O)_5]^{2+}$, PASP–$[Zn(H_2O)_5]^{2+}$ and $[Zn(H_2O)_6]^{2+}$.

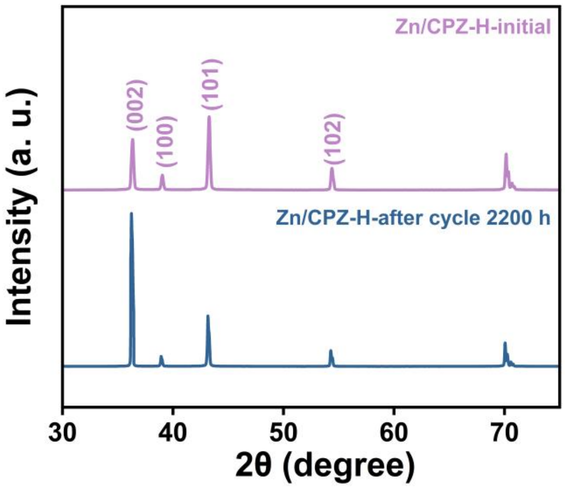

Supplementary Figure 14. XRD patterns of initial Zn/CPZ-H anode and Zn/CPZ-H anode after cycle 2200 h.

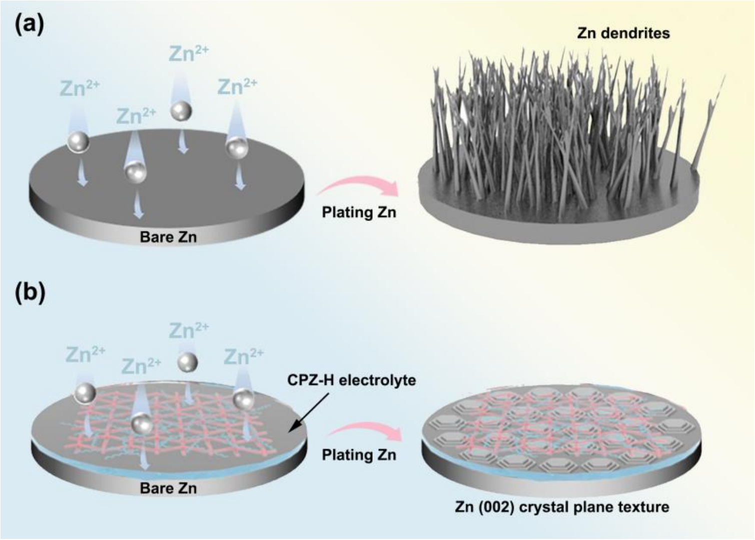

Supplementary Figure 15. Schematic diagram of Zn plating on (a) bare Zn and (b) Zn/CPZ-H anodes.

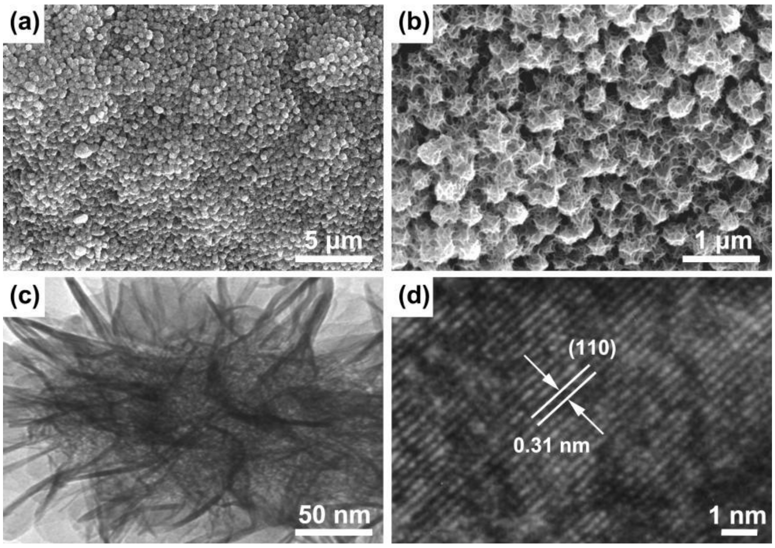

Supplementary Figure 16. Microstructure characterization of MnO_2 cathode. (a,b) SEM images, (c) TEM and (d) HRTEM images of MnO_2 cathode.

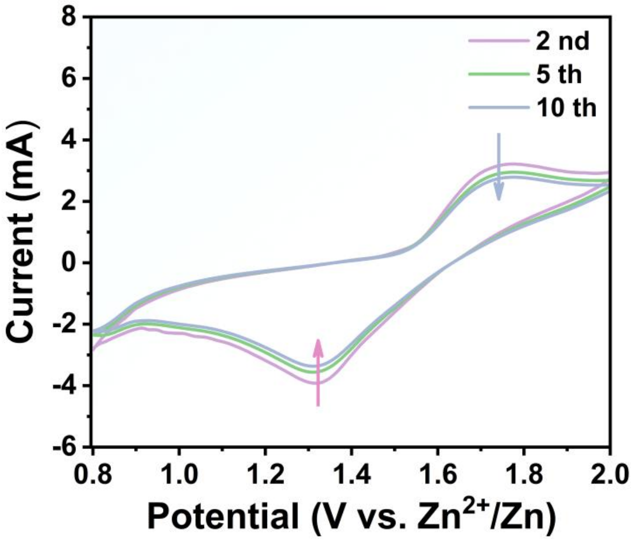

Supplementary Figure 17. CV curves of Zn/CPZ-H//MnO_2 full cell at first 10 cycle.

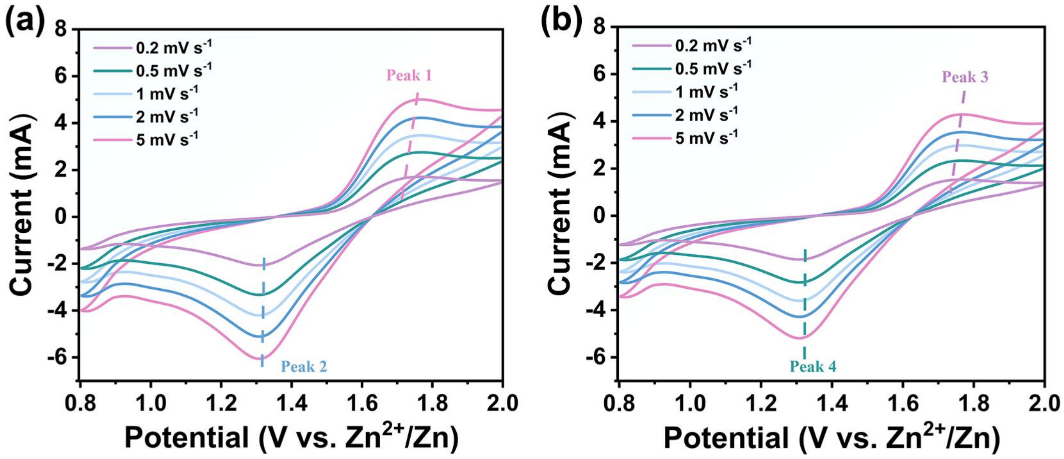

Supplementary Figure 18. CV curves of (a) Zn/CPZ-H//MnO_2 and (b) bare Zn//MnO_2 full cell at scan rate from 0.2 mV s^{-1} to 5 mV s^{-1}.

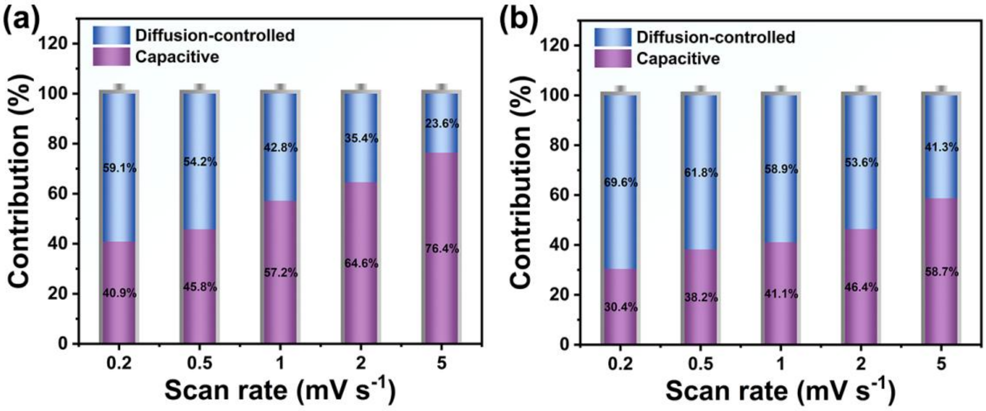

Supplementary Figure 19. Normalized capacity contributions of (a) Zn/CPZ-H// MnO_2 full cell and (b) bare Zn//MnO_2 full cell at different scan rates from 0.2 mV s^{-1} to 5 mV s^{-1}.

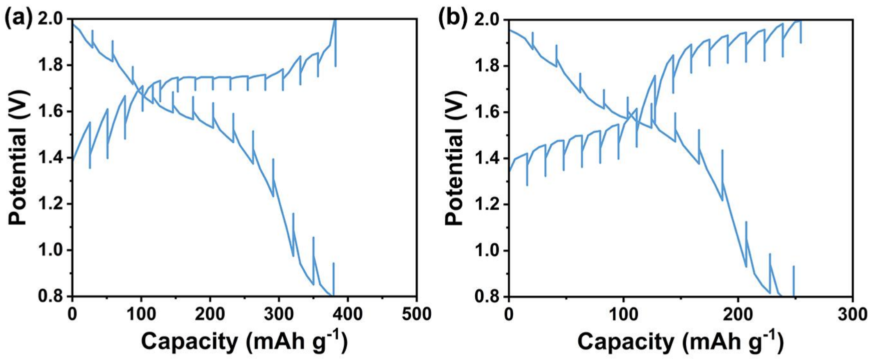

Supplementary Figure 20. GITT curves of (a) Zn/CPZ-H//MnO_2 full cell and (b) bare Zn//MnO_2 full cell at 5 A g^{-1}.

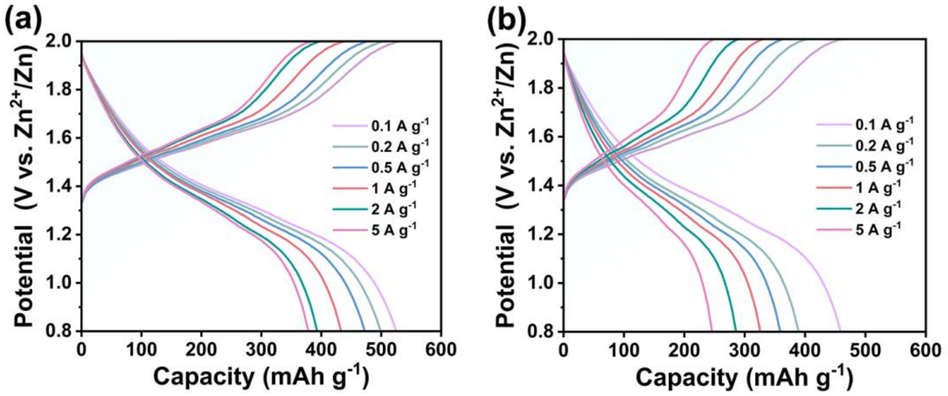

Supplementary Figure 21. GCD curves of (a) Zn/CPZ-H//MnO_2 full cell and (b) bare Zn//MnO_2 full cell at different current densities.

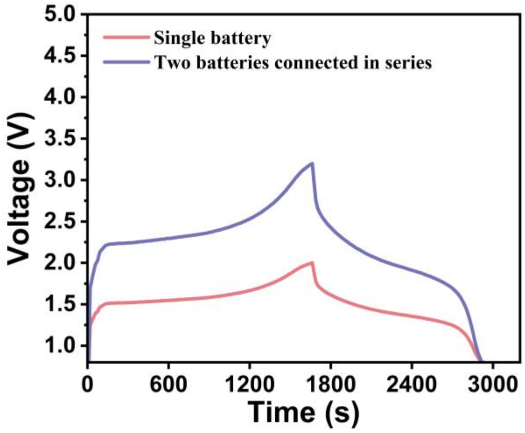

Supplementary Figure 22. GCD curves of single battery and two batteries connected in series.

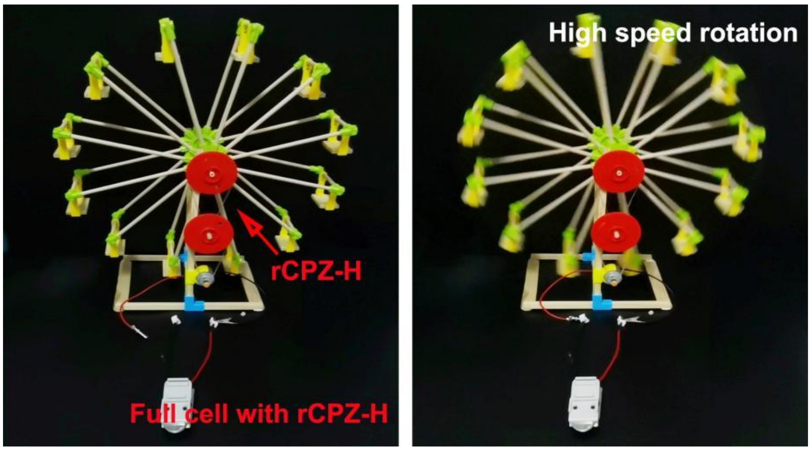

Supplementary Figure 23. Photographs of small Ferris wheel self-powered by Zn/rCPZ-H//MnO_2 full cell.

Supplementary Table 1. Comparison of the assembled Zn/CPZ-H//MnO_2 full cell with the previously reported aqueous Zn ion battery.

Cathode	Anode	Electrolyte	Current Density	Capacity	Life	Energy/Power Density	Reference
3DCEP-MXene/Co-MnHCF	3DCEP-MXene/Zn-P	PAAm-3M $Zn(OTf)_2$	0.2 A g^{-1}	218.4 mAh g^{-1}	95.7% after 1600 cycles at 2 A g^{-1}	283.92 Wh Kg^{-1} 141.96 W Kg^{-1}	[3] Adv. Mater.**2022**, 2209886
$K_2MnFe(CN)_6$	Zn Foil	30 M KFSI+1 M $Zn(CF_3SO_3)_2$	0.2 A g^{-1}	138 mA h g^{-1}	83% after 400 cycles at 0.2 A g^{-1}	150 Wh Kg^{-1} 187.5 W Kg^{-1}	[23] *Adv. Energy Mater.* **2021**, 2003639
V_6O_{13}	ZP@Zn	2M $ZnSO_4$	1 A g^{-1}	326.6 mA h g^{-1}	86.1 % after 100 cycles at 1 A g^{-1}	333.1Wh Kg^{-1} 237.9 W Kg^{-1}	[24] *Angew. Chem. Int. Ed.* **2023**, *e202215324*
$Zn_3V_2O_8$	Zn/PDZ-H	PAAm+DMSO+3 M $Zn(CF_3SO_3)_2$	0.2 A g^{-1}	265.2 mAh g^{-1}	95.27 % After 3000 cycles at 0.2 A g^{-1}	412.3 Wh Kg^{-1} 280.3 Wh Kg^{-1}	[5] Adv. Funct. Mater. **2022**, 2112540
MnO_2	SDF	2 M $ZnSO_4$ and 0.1 M $MnSO_4$	0.3 A g^{-1}	213 mAh g^{-1}	97.6 % after 800 cycles at 3 A g^{-1}	212.81 Wh Kg^{-1} 103.8 W Kg^{-1}	[25] *Adv. Energy Mater.* **2021**, *11, 2100214*
$Cu_3(HHTP)_2$	Zn foil	3M $Zn(CF_3SO_3)_2$	0.1 A g^{-1}	189.2 mAh g^{-1}	75 % after 500 cycles at 4 A g^{-1}	151.4 Wh Kg^{-1} 75.61 W Kg^{-1}	[26] *Nature Communications,* **2019** *10:4948*
$Zn_3V_3O_8$	Zn foil	21 M $LiN(CF_3SO_2)_2$ +1 M $Zn(CF_3SO_3)_2$	0.15 A g^{-1}	127 mAh g^{-1}	72.6 % after 2000 cycles at 5 A g^{-1}	190.5 Wh Kg^{-1} 86.59 W Kg^{-1}	[27] *Energy Storage Materials* **2021** *41 297–309*
MnO_2	PVC-Zn-AAn-COF@Zn	1 M $ZnSO_4$ + 0.1 M $MnSO_4$	0.2 A g^{-1}	302 mAh g^{-1}	73.2 % after 6000 cycles at 2 A g^{-1}	305.02 Wh kg^{-1} 190.6 W kg^{-1}	[28] *Angew. Chem. Int. Ed.* **2022**, *61, e202210871*

$CoFe(CN)_6$	Zn Foil	4 M $Zn(OTf)_2$	0.3 A g^{-1}	173.4 mAh g^{-1}	2200 cycles at 3 A g^{-1}	260.1 Wh Kg^{-1} 104.04 W Kg^{-1}	[29] *Adv. Energy Mater.* **2019**, *1902446*
LMO	PCu@Zn	2 M $ZnSO_4$	0.45 A g^{-1}	84 mAh g^{-1}	77.6 % after 300 cycles at 0.148 A g^{-1}	50.82 Wh Kg^{-1} 23.15 W Kg^{-1}	[17] *Adv. Mater.* **2022**, *34, 2200782*
NVPOF	Zn Foil	0.5 M Zn^{2+} and 1.0 M Na^{+} in TMP	0.2 C	113 mAh g^{-1}	83.5 % after 1000 cycles at 1.0 C	203 Wh Kg^{-1} 145 W Kg^{-1}	[30] *J. Mater. Chem. A*, **2020**, *8, 3252–3261*
VO_2	Zn Foil	PAM-ChNF hydrogel	0.2 A g^{-1}	343.9 mAh g^{-1}	90.7 % after 1000 cycles at 10 A g^{-1}	213.9 Wh Kg^{-1} 139.0 W Kg^{-1}	[31] *Adv. Energy Mater.* **2021**, *2003902*
V_2O_5	3DP-PC/SiOC @Zn	3 M $ZnSO_4$	129.3 mA g^{-1}	0.5 A g^{-1}	76.5 % after 500 cycles at 0.45 A g^{-1}	232.8 Wh Kg^{-1} 155.2 W Kg^{-1}	[32] *Nano Energy* **2020**, *100, 107505*
VO_2	3DP-NC@Zn	1 M $ZnSO_4$	287.9 mA g^{-1}	0.1 A g^{-1}	80 % after 100 cycles at 1 A g^{-1}	273 Wh Kg^{-1} 136.7 W Kg^{-1}	[33] *Adv. Energy Mater.* **2022**, *12, 2103708*
MnO_2	Zn/CPZ-H	CS+PASP+2 M $ZnSO_4$	0.1 A g^{-1}	523.6 mAh g^{-1}	92.5 % after 5000 cycles at 5 A g^{-1}	628.3 Wh Kg^{-1} 392.7 W Kg^{-1} And 452.16 Wh Kg^{-1} 753.6 W Kg^{-1}	Our work
