# Supplementary material for: A recyclable biomass electrolyte towards green zinc-ion batteries
Source: Nat Commun. 2023 Jul 22;14:4435. doi: 10.1038/s41467-023-40178-0 (PMC10363112; doi:10.1038/s41467-023-40178-0)
Supplement: Supplementary file 3 — Description of Additional Supplementary Files [file 41467_2023_40178_MOESM3_ESM.pdf]

Description of Additional Supplementary Files

Supplementary Movie 1. The practical application of the Zn/rCPZ-H//MnO2 full-cell to power small elevator.

Supplementary Movie 2. The practical application of the Zn/rCPZ-H//MnO2 full-cell to maintain high-speed operation of small ferries wheel.
